# Comparison of risk adjustment methods in patients with liver disease using electronic medical record data

**DOI:** 10.1186/s12876-016-0559-4

**Published:** 2017-01-07

**Authors:** Yuan Xu, Ning Li, Mingshan Lu, Elijah Dixon, Robert P. Myers, Rachel J. Jolley, Hude Quan

**Affiliations:** 1 Beijing YouAn Hospital, Capital Medical University, Beijing, China; 2 Department of Community Health Sciences, University of Calgary, Calgary, AB Canada; 3 Department of Economics, University of Calgary, Calgary, AB Canada; 4 Division of General Surgery, Department of Medicine, University of Calgary, Calgary, AB Canada; 5 Liver Unit, Division of Gastroenterology and Hepatology, Department of Medicine, University of Calgary, Calgary, AB Canada

**Keywords:** Risk adjustment, Electronic medical record, Liver disease, In-hospital mortality

## Abstract

**Background:**

Risk adjustment is essential for valid comparison of patients’ health outcomes or performances of health care providers. Several risk adjustment methods for liver diseases are commonly used but the optimal approach is unknown. This study aimed to compare the common risk adjustment methods for predicting in-hospital mortality in cirrhosis patients using electronic medical record (EMR) data.

**Methods:**

The sample was derived from Beijing YouAn hospital between 2010 and 2014. Previously validated EMR extraction methods were applied to define liver disease conditions, Charlson comorbidity index (CCI), Elixhauser comorbidity index (ECI), Child-Turcotte-Pugh (CTP), model for end-stage liver disease (MELD), MELD sodium (MELDNa), and five-variable MELD (5vMELD). The performance of the common risk adjustment models as well as models combining disease severity and comorbidity indexes for predicting in-hospital mortality was compared using c-statistic.

**Results:**

Of 11,121 cirrhotic patients, 69.9% were males and 15.8% age 65 or older. The c-statistics across compared models ranged from 0.785 to 0.887. All models significantly outperformed the baseline model with age, sex, and admission status (c-statistic: 0.628). The c-statistics for the CCI, ECI, MELDNa, and CTP were 0.808, 0.825, 0.849, and 0.851, respectively. The c-statistic was 0.887 for combination of CTP and ECI, and 0.882 for combination of MELDNa score and ECI.

**Conclusions:**

The liver disease severity indexes (i.e., CTP and MELDNa score) outperformed the CCI and ECI for predicting in-hospital mortality among cirrhosis patients using Chinese EMRs. Combining liver disease severity and comorbidities indexes could improve the discrimination power of predicting in-hospital mortality.

## Background

Risk adjustment methods have increasingly been used for a large range of researches, such as health outcomes studies and health care provider performance assessment. In the past few decades, numerous risk adjustment models have been developed for both general medical inpatients as well as disease-specific inpatients, including disease groupers, disease severity indexes, and comorbidity indexes [1]. For liver disease patients, four risk adjustment instruments are commonly used to predict in-hospital mortality: Charlson comorbidity index (CCI) [2], Elixhauser comorbidity index (ECI) [3], Child-Turcotte-Pugh (CTP) [4, 5], and model for end-stage liver disease (MELD) [6, 7].

CCI was originally developed based on medical charts to estimate 1-year mortality of patients with breast cancer and was validated in another 10-year follow-up cohort [3]. Since then, the index has been most widely used for risk adjustment [8, 9]. In 1998 Elixhauser et al. introduced a new comorbidity algorithm based on United States administrative health data to define the 31 conditions for predicting health outcomes including in-hospital mortality, hospital cost and length of stay [3]. Liver disease studies have shown that ECI performed better than CCI in administrative health data [9–12].

Hepatologists use CTP or MELD frequently to predict short-term prognoses or outcomes such as in-hospital mortality, post-surgery mortality or procedure related complications in patients with chronic liver diseases given both instruments are readily applicable at bedside. CTP contains five clinical measures and could be used either as CTP classifications (3 classes for 10 levels of risk) or as a summary score. CTP included two subjective measures (degree of ascites and encephalopathy) which lead to the issue of inter-rater variation. MELD score, on the other hand, does not employ any subjective measure and includes three laboratory test results instead. MELD was initially used to evaluate the risk of death after transjugular intrahepatic portosystemic shunt for patients with cirrhosis, and later used for predicting mortality before or after liver transplantation for patients with end-stage liver diseases [6, 7]. MELD score is regarded more objective and reproducible than CTP and replaced CTP in organ allocation systems such as United Network for Organ Sharing for patients waiting for liver transplantation [13, 14].

The choice of these four risk adjustment methods often depends on data availability. CCI and ECI can be constructed using administrative health data [9, 10, 12, 15–19], while MELD and CTP are used in primary clinical data [20–24]. As a result, performance of these risk adjustment models has not been compared on the same liver patient population. It remains unclear what is the best risk adjustment approach for liver disease.

As a result of rapid development and wide use of electronic medical record (EMR) in China in recent years, an enormous amount of EMR data is being collected [25, 26]. Additionally, liver diseases, including viral hepatitis, cirrhosis, and primary liver cancer (PLC), are highly prevalent in China [27]. About 97 million people are hepatitis B carriers [28]; at least 20 million patients have chronic hepatitis B with or without cirrhosis and/or PLC [27, 28]. Between 2006 and 2010, about 1.2% of inpatients in general hospitals in Beijing were admitted due to cirrhosis (mainly hepatitis cirrhosis) [29]. Therefore, Chinese hospital EMR data provides a unique chance to conduct the comparison study of different risk adjustment methods in the content of liver disease.

To the best of our knowledge, this is the first study that compares the performance of common risk adjustment models in predicting in-hospital mortality for the same large inpatient population with cirrhosis.

## Methods

### Data source and study population

The data used in our study was derived from the EMR of Beijing YouAn hospital, one of the leading teaching hospitals specialized in liver diseases in China and treating over 300,000 patients from all over China each year. In 2008, the EMR system was officially implemented in YouAn hospital and inpatient documentation completely switched from paper charts to EMR. For each patient, the EMR contains a front summary page, as well as sections with detailed information on admission, discharge, surgery/procedure, death, laboratory test results, radiology test results, pathology report, physician’s notes, hospitalization billing records, and electronic prescription. Among these sections, laboratory test results, electronic prescriptions, and billing records are completely structured without any free text. The front page, admission and discharge records, and radiology test results, however, are only semi-structured and contain both structured drop-down lists and free-text fields. The hospital assigned a unique identification number to each patient; all sections of EMRs are linked using the identification number.

The study population included patients with cirrhosis hospitalized at Beijing YouAn hospital between January 1st, 2010 and September 30th, 2014, who were at least 18 years old and consented to use their EMRs for research (nearly all patients provided consent), and excluded patients with missing in-hospital mortality status. We excluded 145 (1.3%) patients due to missing information on in-hospital mortality (the missing was likely caused by physicians’ unintentional incomplete documentation) and 180 (1.6%) patients who underwent liver transplantation, given this group of patients were much more complicated in contrast to other patients. In total, 11,122 adult cirrhosis patients were analyzed. This study was approved by the YouAn Hospital Research Board of Ethics and the Health Research Ethics Board at University of Calgary (Ethic committee’s reference number: REB14-0815).

### Outcome and independent variables

The outcome measure was in-hospital mortality that was recorded in the EMR. Liver disease variables were defined using our previously developed and validated EMR case definitions [30]. The validation study showed that most of the case definitions had high validity (positive predictive value over 80%). Using the validated case definitions, we defined the following variables: cirrhosis, PLC, hepatitis, hepatic encephalopathy (HE) and ascites, as well as the Charlson and Elixhauser comorbidities at the time of admission. In addition, the laboratory test results required to construct the CTP and MELD scores were directly extracted from the EMR system. These laboratory test results included the serum level of albumin, total bilirubin, creatinine, sodium (Na), and the international normalized ratio of prothrombin time (PT-INR). For inpatient episode with multiple laboratory tests, results from the tests conducted at or immediately after admission were used. Only 232 patients had missing values of one of above laboratory tests. We assumed these missing values fell in the normal range at admission. Chart review on 30 charts randomly selected out of these 232 patients supported this assumption.

Using EMR data in the latest admission, we defined in-hospital mortality and the laboratory test results. To define chronic diseases (e.g., comorbidities) we included the information in the multiple admissions within 1 year prior to the latest admission date.

### Risk adjustment models

Commonly used variants of CCI [10, 12] were tested: the all individual comorbidities of CCI (referred to as CCI), the number of Charlson comorbidities categorized (0, 1, 2, ≥3 comorbidities) (referred to as CCI categorized), the score of CCI (referred to as CCI score), which is the summation of the weighted score of each comorbidity, and the categorized CCI score (0, 1–2, 3–4, ≥5 points) (referred to as CCI score categorized), (See detailed description of the tested models in Table 1). For ECI, models using the individual Elixhauser comorbidities (referred to as ECI), and the number of Elixhauser comorbidities categorized (0, 1, 2, ≥3 comorbidities) (referred to as ECI categorized) [11] were tested (Table 1). Both CCI and ECI contain variables related to liver diseases. We excluded “mild/moderate to severe liver disease” in CCI and the “liver disease” in ECI. PLC was excluded from the variables of “any malignancy”, “metastatic solid tumor”, and “solid tumor without metastases”.

**Table 1:** The description of compared models

Method		Variants	Description
Comorbidity methods	CCI	CCI	Charlson individual comorbidities (binary variables)
		CCI categorized	number of Charlson comorbidities excluding liver disease (0, 1, 2, ≥3)
		CCI score	the score of Charlson comorbidities (weighted score)
		CCI score categorized	the score of CCI categorized as 0, 1–2, 3–4, ≥5
	ECI	ECI	Elixhauser individual comorbidities (binary variables)
		ECI categorized	number of Elixhauser comorbidities (0, 1, 2, ≥3)
Liver specific severity methods	MELD	MELD score	calculated by total bilirubin, PT-INR, and creatinine
		MELDNa score	calculated by MELD score and serum sodium
		5vMELD score	calculated by MELDNa score and serum albumin
	CTP	CTP	the classification of Child-Turcotte-Pugh (including the individual binary variables of hepatic encephalopathy, ascites, total bilirubin, prothrombin time, and albumin)
		CTP score	calculated by summing the weighted score of each CTP variable
Comorbidity + Liver severity		MELDNa score + ECI	the score of MELDNa + individual binary variable of Elixhauser comorbidities
		CTP + ECI	individual binary variables of CTP + individual binary variable of Elixhauser comorbidities

*CTP* Child-Turcotte-Pugh, *MELD* model for end-stage liver disease, *MELDNa* MELD sodium, *5vMELD* five variable MELD, *CCI* Charlson comorbidity index, *ECI* Elixhauser comorbidity index

For MELD, three common variants were tested (Table 1), including MELD score (referred to as MELD score) [6], MELD sodium score (referred to as MELDNa score) [31], and five-variable MELD score (referred to as 5vMELD score) [32]. MELD score = 3.78 × ln[serum total bilirubin (mg/dL)] + 11.2 × ln[INR] + 9.57 × ln[serum creatinine (mg/dL)] + 6.43) [6]. To avoid scores below 0 in the logarithm, value less than one is rounded to 1 (e.g., for total bilirubin with 0.75, a value of 1.0 is assigned). MELDNa = MELD score + 1.59 [135 - Na], where Na is bounded between 120 and 135 mmol/L (Na lower than the low limit is assigned with a value of 120 mmol/L, and Na higher than 135 mmol/L is assigned a value of 135 mmol/L) [31]. 5vMELD score = MELDNa + (5.275 × [4-albumin]) – (0.136 × MELDNa × [4 - serum albumin]), where albumin is bounded between 1 and 4 g/dL [32]. Two variants of CTP were tested: CTP classification (referred to as CTP) and CTP score (referred to as CTP score) (Table 1). The CTP score is defined by summing the assigned score for each of the five variables including HE (absence = 1, slight-medium = 2, and refractory = 3), ascites (none = 1, mild = 2, and moderate to severe = 3), total bilirubin (<34 μmol/L = 1, 34–50 μmol/L = 2, and >50 μmol/L = 3), PT-INR (<1.7 = 1, 1.7–2.3 = 2, and > 2.3 = 3), and albumin (>3.5 = 1, 2.8–3.5 = 2, and <2.8 = 3) [5]. Calculating CTP score requires the refined severity of HE and ascites; however, 13.5% patients had unknown severity of HE, and the patients with unknown severity of ascites accounted for 52.6%. We excluded these patients from the CTP score model because we were not able to calculate CTP score for these patients. To include the patients with unknown severity of HE or ascites, we also categorized HE and ascites into binary variables (presence or absence) in the CTP classification model. In addition, we tested risk adjustment models using combination of CTP, MELDNa scorer and ECI (Table 1). For these models incorporated both comorbidity index and liver disease severity score, we tested the interactions between different risk adjustment instruments.

### Statistical analysis

Descriptive analysis was conducted and logistic regression models (as described above) were used to predict in-hospital mortality. The baseline model consisted of age, sex, and admission status (urgently or not). Concordance-statistic (c-statistic) was used to assess the performance of the risk adjustment models [33, 34]. C-statistic of 0.5 means that the ability of discrimination of the model is zero; the discrimination power is regarded as “unacceptable” when c-statistic range from 0.50 to 0.69; or “acceptable” when c-statistic range from 0.70 to 0.79; or “good to excellent” when c-statistic is 0.80 or greater. The 10-fold cross validation [35] was used to calculate the corrected c-statistics to adjust for the number of independent variables in the model considering that c-statistic increases with the number of independent variables. We also conducted bootstrapping (1000 samples) and calculated 95% confidence interval for c-statistics (95% CI) for internal validation of the c-statistic of each model [36].

Probability of death for each patient was calculated by the logistic regression models; patients were ranked and allocated to different risk groups based on the predicted probability of death. The agreement of observed and expected number of death was assessed. Graphs were plotted to show the expected and observed mortality rates across the various risk groups.

In addition, similar analyses were conducted using the subsample of patients with viral hepatitis, alcoholic hepatitis, PLC, decompensated cirrhosis, and no-procedure subgroups (without undergoing hepatectomy, liver transplantations, transcatheter arterial chemoembolization, and endoscopic treatment). All analyses were performed in SAS version 9.4 (Cary, NC).

## Results

Of 11,121 cirrhotic patients (Table 2), the median age was 53 (interquartile range: 46–61) years, 69.9% (7773) were male and 11.0% (1219) patients were admitted emergently. The common causes for cirrhosis were hepatitis B (73.1%), alcoholic hepatitis (25.0%), hepatitis C (8.8%), and fatty liver (4.6%). Of the cirrhosis patients, 3824 (34.4%) had PLC (hepatocellular carcinoma account for 96.5%); and 5433 (48.9%) patients did not undergo any major surgeries or procedures (i.e., hepatectomy, liver transplantation, transcatheter arterial chemoembolization, sclerotherapy and variceal banding), radiofrequency ablation or radiotherapy. Overall the in-hospital mortality was 8.3%.

**Table 2:** Characteristics of patients with cirrhosis (*N* = 11,121)

Characteristics	Median (interquartile range) or frequency
Na (mmol/L)	138.4 (135.0–140.0)
Creatinine (umol/L)	66.3 (54.5–81.9)
PT-INR	1.2 (1.0–1.4)
Total bilirubin (umol/L)	27.2 (17.1–59.3)
Albumin (g/dl)	34.7 (29.4–40.0)
CTP score^a^	5.0 (5.0–6.0)
MELD score	8.0 (7.0–11.0)
MELDNa score	10.0 (8.0–14.0)
5vMELD score	13.0 (9.0–18.0)
Charlson comorbidity score	1 (0–2)
LOS (day)	13 (5–26)
Age (year)	
18–44	2576 (23.2%)
45–64	6785 (61.0%)
≥ 65	1761 (15.8%)
Male	7773 (69.9%)
Urgent admission	1219 (11.0%)
Na < 135 mmol/L	2764 (24.9%)
Creatinine > 88.4 umol/L	2190 (19.7%)
PT-INR	
< 1.7 (normal range)	10071 (90.6%)
1.7–2.2	724 (6.5%)
> 2.2	327 (2.9%)
Total bilirubin (umol/L)	
< 34.2 (normal range)	6564 (59.0%)
34.2–51.3	1328 (11.9%)
> 51.3	3230 (29.0%)
Albumin (g/dl)	
> 3.5 (normal range)	5370 (48.3%)
2.8–3.5	3660 (32.9%)
< 2.8	2092 (18.8%)
Hepatic encephalopathy^b^	
Grade I-II	455 (4.1%)
Grade III-IV (or refractory)	109 (1.0%)
Severity unknown	1505 (13.5%)
Ascites^b^	
Mild	522 (4.7%)
Moderate to sever	103 (0.9%)
Severity unknown	5853 (52.6%)
CTP classification^b^	
A (CTP score 5–6)	3952 (35.5%)
B (CTP score 7–9)	1002 (9.0%)
C (CTP score 10–15)	80 (0.7%)
Number of abnormal CTP variables	
0	2803 (25.2%)
1	2255 (20.3%)
2	2390 (21.5%)
≥ 3	3674 (33.0%)
Number of Charlson comorbidities	
0	3580 (32.2%)
1	4564 (41.0%)
2	2212 (19.9%)
≥ 3	766 (6.9%)
Number of Elixhauser comorbidities	
0	2595 (23.3%)
1	3239 (29.1%)
2	2355 (21.2%)
≥ 3	2933 (26.4%)
Charlson comorbidities	
Myocardial infarction	62 (0.6%)
Cerebrovascular disease	371 (3.3%)
Dementia	6 (0.1%)
Renal disease	1228 (11.0%)
Any malignancy^c^	645 (5.8%)
Charlson and Elixhauser shared comorbidities	
Congestive heart failure	25 (0.2%)
Peripheral vascular disease	7 (0.1%)
Chronic pulmonary disease	191 (1.7%)
Rheumatologic disease	62 (0.6%)
Peptic ulcer disease	1234 (11.1%)
Diabetes complicated	130 (1.2%)
Diabetes uncomplicated	3957 (35.6%)
Hemiplegia or paraplegia	8 (0.1%)
Metastatic solid tumor^c^	275 (2.5%)
AIDS	40 (0.4%)
Elixhauser comorbidities^d^	
Cardiac arrhythmias	447 (4.0%)
Valvular disease	30 (0.3%)
Hypertension uncomplicated	2008 (18.1%)
Hypertension complicated	1156 (10.4%)
Hypothyroidism	95 (0.9%)
Lymphoma	20 (0.2%)
Solid tumor without metastasis^c^	178 (1.6%)
Coagulopathy	5 (0.04%)
Blood loss anemia	335 (3.0%)
Deficiency anemia	959 (8.6%)
Depression	41 (0.4%)
Fluid and electrolyte disorders	1707 (15.4%)
Alcohol abuse	2780 (25.0%)
Psychoses	19 (0.2%)
Renal failure	236 (2.1%)

*IQR* interquartile range, *Na* serum sodium, *PT-INR* international normalized ratio of prothrombin time, *LOS* length of stay in hospital, *CTP* Child-Turcotte-Pugh, *MELD* model for end-stage liver disease, *MELDNa* MELD sodium, *5vMELD* five variable MELD, *AIDS* acquired immune deficiency syndrome

^a^CTP score was not available for patients with unknown severity of HE or ascites

^b^The sum of proportion of the categories is less than 100% because there were missing values on acsites and hepatic encephalopathy

^c^Excluded primary liver cancer

^d^The obesity, weight loss, pulmonary circulatory disorders, other neurological disorders and drug abuse were excluded due to 0% prevalence

### Outcome measure and independent variables

At time of admission, 25.0% (2764) of the cirrhotic patients were diagnosed with hyponatremia (Na < 135 mmol/L), 19.7% (2190) with high creatinine level (>88.4 umol/L), 9.5% (1051) with abnormal PT-INR (>1.7), 40.98% (4558) with high total bilirubin level (>34.2 umol/L), and 51.7% (5752) with hypoproteinemia (albumin < 2.8 g/dL). At time of admission, 18.6% (2069) of the cirrhotic patients had HE, and 58.3% (6478) had ascites. The most common five comorbidities were diabetes uncomplicated (35.6%), hypertension (complicated and uncomplicated) (28.5%), alcohol abuse (25.0%), fluid and electrolyte disorder (15.4%) and peptic ulcer disease (11.1%).

In general, in-hospital mortality was higher among male patients, older patients, urgently admitted patients, patients with abnormal clinical variables, patients with a certain comorbidity (except for acquired immune deficiency syndrome and peripheral vascular disease), or patients with higher MELD, MELDNa or 5vMELD score than their counterparts (see Table 3). As number of Charlson or Elixhauser comorbitites increased, so did in-hospital mortality. A similar pattern was found with the number of abnormal CTP variables.

**Table 3:** Crude in-hospital mortality by study variables (*N* = 11,121)

Variables		Mortality % (n)	*P*-value^1^
Age (year)	18–44	4.9 (125)	<0.0001
	45–64	8.0 (543)	
	≥65	14.3 (252)	
Sex	male	9.1 (704)	<0.0001
	female	6.5 (216)	
Admission status	non-urgent	6.3 (627)	<0.0001
	urgent	24.0 (293)	
Hepatic encephalopathy	no	4.0 (360)	<0.0001
	yes	27.1 (560)	
Ascites	no	1.8 (85)	<0.0001
	yes	12.9 (835)	
PT-INR	<1.7	6.2 (628)	<0.0001
	1.7–2.3	23.2 (168)	
	>2.3	37.9 (124)	
Total bilirubin (umol/L)	<34.2	4.1 (271)	<0.0001
	34.2–51.3	7.1 (94)	
	>51.3	17.2 (555)	
Albumin (g/dl)	>35	3.2 (170)	<0.0001
	28–35	10.0 (367)	
	<28	18.3 (383)	
Creatinine (umol/L)	≤88.4	5.0 (445)	<0.0001
	>88.4	21.7 (475)	
Na	≥135	4.4 (364)	<0.0001
	<135	20.1 (556)	
MELD score	<7.0	1.9 (54)	<0.0001
	7.0–8.0	3.6 (100)	
	8.0–10.0	7.0 (195)	
	>10.0	20.5 (571)	
MELDNa score	<8.0	0.7 (19)	<0.0001
	8.0–10.0	2.7 (74)	
	10.0–14.0	6.6 (184)	
	>14.0	23.1 (643)	
5vMELD score	<9.0	0.5 (15)	<0.0001
	9.0–13.0	2.3 (65)	
	13.0–18.0	8.0 (223)	
	>18.0	22.2 (617)	
CTP classification	A (CTP score 5–6)	0.9 (34)	<0.0001
	B (CTP score 7–9)	3.2 (32)	
	C (CTP score 10–15)	11.3 (9)	
Number of abnormal CTP variables	0	0.5 (13)	<0.0001
	1	2.3 (52)	
	2	6.2 (147)	
	≥3	19.3 (708)	
Myocardial infarction	no	8.2 (902)	<0.0001
	yes	29.0 (18)	
Cerebrovascular disease	no	7.9 (846)	<0.0001
	yes	20.0 (74)	
Renal disease	no	5.9 (586)	<0.0001
	yes	27.2 (334)	
Any malignancy^a^	no	8.0 (841)	<0.0001
	yes	12.2 (79)	
Congestive heart failure	no	8.3 (10)	<0.0001
	yes	40.0 (10)	
Chronic pulmonary disease	no	8.1 (888)	<0.0001
	yes	16.8 (32)	
Rheumatologic disease	no	8.3 (913)	0.39
	yes	11.3 (7)	
Peptic ulcer disease	no	8.1 (801)	0.06
	yes	9.6 (119)	
Diabetes complicated	no	8.3 (907)	0.47
	yes	10.0 (13)	
Diabetes uncomplicated	no	5.2 (374)	<0.0001
	yes	13.8 (546)	
Hemiplegia or paraplegia	no	8.3 (919)	0.66
	yes	12.5 (1)	
Metastatic solid tumor	no	7.8 (844)	<0.0001
	yes	27.6 (76)	
Cardiac arrhythmias	no	7.9 (840)	<0.0001
	yes	17.9 (80)	
Valvular disease	no	8.3 (916)	0.31
	yes	13.3 (4)	
Hypertension uncomplicated	no	7.8 (708)	<0.0001
	yes	10.6 (212)	
Hypertension complicated	no	7.9 (784)	<0.0001
	yes	11.8 (136)	
Hypothyroidism	no	8.3 (911)	0.67
	yes	9.5 (9)	
Lymphoma	no	8.3 (918)	0.78
	yes	10.0 (2)	
Solid tumor without metastasis^a^	no	8.1 (886)	0.31
	yes	19.2 (34)	
Coagulopathy	no	8.3 (918)	0.01
	yes	40.0 (2)	
Blood loss anemia	no	7.6 (818)	<0.0001
	yes	30.5 (102)	
Deficiency anemia	no	7.4 (755)	<0.0001
	yes	17.2 (165)	
Depression	no	8.2 (913)	<0.0001
	yes	17.1 (7)	
Fluid and electrolyte disorders	no	5.5 (519)	<0.0001
	yes	23.5 (401)	
Psychoses	no	8.3 (918)	0.72
	yes	10.5 (2)	
Renal failure	no	7.4 (803)	<0.0001
	yes	49.6 (117)	
AIDS	no	8.3 (920)	0.06
	yes	0.0 (0)	
Peripheral vascular disease	no	8.3 (920)	0.43
	yes	0.0 (0)	
Alcohol abuse	no	7.49 (625)	<0.0001
	yes	10.62 (295)	
Number of Charlson comorbidities	0	2.6 (92)	<0.0001
	1	6.0 (272)	
	2	14.2 (315)	
	≥3	31.5 (241)	
Charlson comorbidity score categorized	0	2.6 (92)	<0.0001
	1–2	6.2 (310)	
	3–4	16.75 (325)	
	≥5	30.4 (193)	
Number of Elixhauser comorbidities	0	2.1 (54)	<0.0001
	1	3.9 (126)	
	2	7.4 (175)	
	≥3	19.3 (565)	

*IQR* interquartile range, *Na* serum sodium, *PT-INR* International normalized ratio of prothrombin time, *CTP* Child-Turcotte-Pugh, *MELD* model for end-stage liver disease, *MELDNa* MELD sodium, *5vMELD* five variable MELD, *AIDS* acquired immune deficiency syndrome

^1^ *P*-value of Chi-square exact test is for each contingency table (mortality by each predictor)

^a^Excluded primary liver cancer

### Performance of risk adjustment models

The c-statistics and its 95% confidence intervals (CI) of the risk adjustment models predicting in-hospital mortality for overall cirrhotic patients were presented in Table 4, while those for the subgroups of cirrhotic patients (viral hepatitis, alcoholic hepatitis, PLC, decompensated cirrhosis, and non-procedure) were presented in Table 5.

**Table 4:** C-statistics (95% CI) for predicting in-hospital mortality of the compared risk adjustment methods^a^ in the overall cirrhosis patients (*N* = 11,121)

Model	Mean c-statistic (95% CI)^b^	Bias-corrected c-statistic^c^
CCI	0.809 (0.792–0.822)	0.816
CCI categorized	0.786 (0.771–0.801)	0.784
CCI score	0.785 (0.769–0.799)	0.787
CCI score categorized	0.786 (0.770–0.801)	0.783
ECI	0.825 (0.749–0.848)	0.827
ECI categorized	0.794 (0.743–0.841)	0.773
MELDNa score	0.849 (0.838–0.861)	0.849
5vMELD score	0.845 (0.833–0.858)	0.847
MELD score	0.818 (0.805–0.833)	0.817
CTP	0.851 (0.839–0.864)	0.847
CTP score	0.793 (0.736–0.844)	0.803
MELDNa score + ECI	0.882 (0.826–0.898)	0.882
CTP + ECI	0.887 (0.846–0.901)	0.885

*CI* confidence interval, *CCI* Charlson comorbidity index, *ECI* Elixhauser comorbidity index, *CTP* Child-Turcotte-Pugh, *MELD* model for end-stage liver disease, *MELDNa* MELD sodium, *5vMELD* five variable MELD

^a^Age, sex and admission status were included in all regression models

^b^1000 samples bootstrapping mean c-statistic and 95% CI

^c^10-fold cross validation corrected c-statistic

**Table 5:** C-statistics (95% CI)^a^ of the logistic regression models^b^ in the subgroups of cirrhotic patients

Model	Viral hepatitis	PLC	Alcoholic hepatitis	Decompensated	No-procedure^c^
Number of cases (%)	8132 (73.1)	3824 (34.4)	2778 (25.0)	7183 (64.6)	5433 (48.9)
CCI	0.807 (0.789–0.825)	0.788 (0.764–0.812)	0.791 (0.761–0.817)	0.788 (0.771–0.805)	0.796 (0.778–0.815)
ECI	0.821 (0.756–0.846)	0.828 (0.805–0.849)	0.837 (0.809–0.860)	0.807 (0.786–0.825)	0.834 (0.817–0.852)
MELDNa score	0.848 (0.835–0.863)	0.846 (0.828–0.861)	0.853 (0.833–0.873)	0.827 (0.810–0.841)	0.845 (0.831–0.860)
CTP	0.856 (0.842–0.869)	0.869 (0.851–0.885)	0.847 (0.827–0.867)	0.822 (0.806–0.838)	0.852 (0.837–0.867)
MELDNa score + ECI	0.878 (0.836–0.897)	0.888 (0.871–0.903)	0.896 (0.876–0.914)	0.863 (0.846–0.877)	0.889 (0.876–0.902)
CTP + ECI	0.887 (0.847–0.905)	0.907 (0.891–0.920)	0.897 (0.878–0.914)	0.864 (0.848–0.877)	0.892 (0.878–0.904)

*CI* confidence interval, *CCI* Charlson comorbidity index, *ECI* Elixhauser comorbidity index

*CTP* Child-Turcotte-Pugh, *MELDNa* model for end-stage liver disease and sodium

^a^1000 samples bootstrapping mean c-statistic and 95% CI

^b^Age, sex and admission status were included in all regression models

^c^Procedure refers to the major procedures such as the hepatectomy, liver transplantation, transcatheter arterial chemoembolization, endoscopic treatment (i.e., sclerotherapy and variceal banding), and radiofrequency ablation and radiotherapy

For model with age, sex and admission status as the baseline model, c-statistic was 0.628 (95% CI: 0.609–0.650). All risk adjustment models with comorbidities, MELD or CTP significantly outperformed the baseline model, with c-statistics ranging from 0.785 to 0.887. For models with variable of the number of comorbidities (0, 1, 2 and ≥3), the c-statistic obviously dropped from 0.825 (95% CI: 0.749–0.848) to 0.794 (95% CI: 0.743–0.841) for ECI; and from 0.809 (95% CI: 0.792–0.822) to 0.786 (95% CI: 0.771–0.801) for CCI. The CCI score categorized model had very similar c-statistic with the CCI score model (0.786 versus 0.785). The c-statistic for MELD score model (0.818, 95% CI: 0.805–0.833) was significantly lower than MELDNa score model (0.849, 95% CI: 0.838–0.861) and 5vMELD score model (0.845, 95% CI: 0.833–0.858). The performance of the CTP is very similar with the MELDNa score (c-statistics 0.851 versus 0.849, *p* = 0.073). The performance of CTP score was significantly lower than CTP (c-statistics: 0.793, 95% CI: 0.736–0.844 versus 0.851, 95% CI: 0.839–0.864). In summary, for the overall cirrhotic patients, among the risk adjustment models, c-statistics increased in a consistent order from the CCI, ECI, MELDNa score, to CTP. The comparison result using bias-corrected c-statistic was slightly different (order from low to high performance: CCI, ECI, CTP to MELDNa score). The corrected c-statistics for CTP and MELDNa score models were very similar (0.847 versus 0.849).

Results on model performance within patient’s subgroups (those with viral hepatitis, alcoholic hepatitis, PLC, decompensated cirrhosis, and no-procedure subgroups) remained the same: c-statistics increased in a consistent order from the CCI, ECI to MELDNa score (or CTP). Compared with models employing only single risk adjustment model, c-statistic of models that combined both liver disease severity and comorbidity indexes was shown to be better. Model combining CTP and ECI improved the c-statistic compared with the CTP model (c-statistics: 0.887 versus 0.851, *p* < 0.0001). Similarly, model that combined ECI and MELDNa score outperformed model that includd MELDNa score only (c-statistics: 0.882 versus 0.849, *p* < 0.0001).

**Fig. 1:**
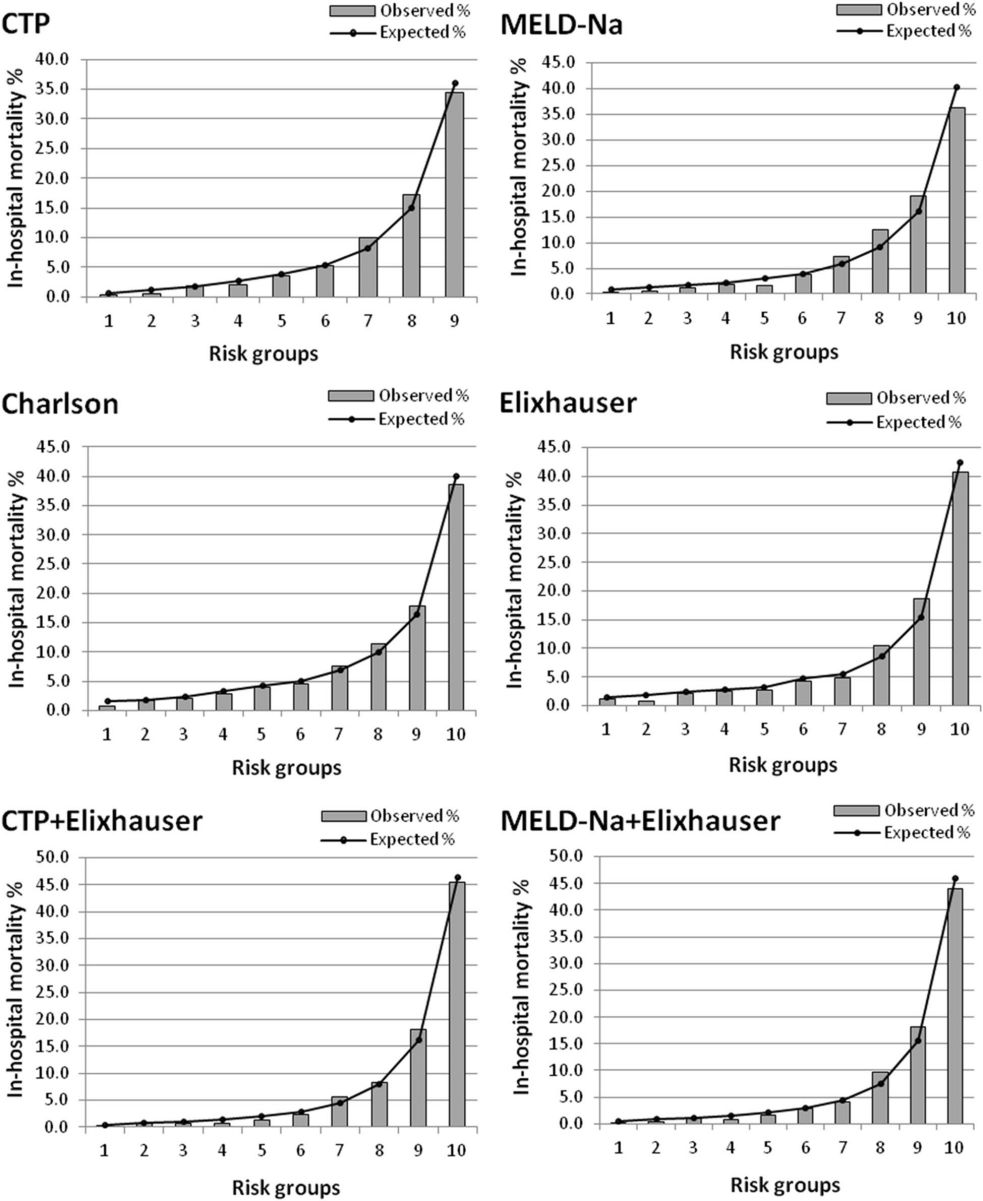
Expected and observed mortality in various risk groups for patients with cirrhosis. CTP: Child-Turcotte-Pugh; MELDNa: model for end-stage liver disease and sodium

Figure 1 presents the observed and expected mortality across model-defined risk groups for the six models (CCI, ECI, MELDNa score, CTP, ECI + MELDNa score, and ECI + CTP) in the overall sample. The “spread-out” of the expected mortality generated from combined models (i.e., CI + MELDNa score and ECI + CTP) was much wider than the models with only comorbidities, MELD or CTP.

## Discussion

To the best of our knowledge, this is the first study that compared the performance of common risk adjustment methods in predicting in-hospital mortality for patients with cirrhosis, using large Chinese EMR data. The EMR data provided comprehensive information on both comorbidities as well as disease specific clinical information for large inpatient sample, presenting researchers a valuable opportunity to assess performance of various risk adjustment models on the same patient population. Our large sample also statistically empowered precision of the assessment. Overall, our study highlighted: 1) liver specific scores of CTP and MELDNa performed better than comorbidity methods of CCI and ECI; 2) combination of liver disease severity and comorbidity indexes (such as CTP + ECI or MELDNa score + ECI) significantly improved performance of in-hospital mortality prediction; and 3) these findings were consistent across subtypes of liver diseases.

### Comparison of risk adjustment methods

We assessed the performance of risk adjustment models in predicting in-hospital mortality for patients with cirrhosis, using a single model or a combination of two models among ECI and CTP (or MELDNa score). All models significantly outperformed the baseline model with age, sex, and admission status. These results provided support of the use of these models as risk adjustment instruments for liver disease. While all models were shown to have reasonable predictive power, liver disease severity indexes (CTP and MELDNa score) were shown to be better than the comorbidity indexes (CCI and ECI). Moreover, comparing with individual comorbidity or liver disease severity index, combined models (e.g., CTP + ECI or MELDNa + ECI) demonstrated higher performance in predicting in-hospital mortality.

Between the two comorbidity indexes tested, ECI was found to be more predictive than CCI among all cirrhotic patients as well as for all the subgroups. This result was consistent with findings in the existing risk adjustment literatures for liver disease that used administrative data [9, 16, 18, 37]. The better performance of ECI could be explained by that ECI identified substantially more conditions than CCI, which contributed to a higher c-statistic [16, 37]. In our study, we used a category of number of comorbidities presence as one independent variable for ECI and CCI. This method showed similar c-statistics for ECI and CCI (0.794 versus 0.786).

Among the liver disease severity indexes tested, the discrimination ability of CTP was consistently shown to be higher than MELD and 5vMELD scores, and close to MELDNa score among all of the subgroups. This proved the appropriateness of ongoing use of CTP in practice to predict in-hospital mortality in cirrhotic patients. However, refined degree of HE and ascites may not be available in many datasets, making it impossible to use CTP as a risk adjustment instrument. The construction of MELDNa score only requires routine laboratory test results, which makes MELDNa score more reproducible, reliable and easier to apply [20–22]. More importantly, our results showed that the performance of MELDNa score were very close to or better than that of CTP. This indicates that using MELDNa score instead of CTP might simplify the analysis without compromising the predictive accuracy.

MELDNa and 5vMELD scores had similar performance in predicting in-hospital mortality. 5vMELD score was generated through adding serum albumin level to MELDNa score. The additional variable in 5vMELD did not significantly improve its predictability of in-hospital mortality. The possible reason is that albumin level measured during hospitalization did not reflect the patient’s severity of disease because albumin was commonly administrated in inpatients with cirrhosis.

Overall, the liver disease severity indexes (MELD score and CTP) outperformed the comorbidity indexes (CCI and ECI) on prediction of in-hospital mortality. The possible reason is that the most recent laboratory test results within one hospitalization episode could reflect the severity of liver disease at the occurrence of hospitalization outcome (mortality). We conducted sensitivity analysis to address this explanation. We calculated MELDNa score at near discharge time and fitted model to predict in-hospital mortality. The c-statistic for MELDNa model increased significantly from 0.849 (95% CI: 0.838–0.861) for near admission time to 0.912 (95% CI: 0.903–0.921) for near discharge time. This supported our hypothesis that the performance of risk adjustment instruments improves when they are constructed based on information collected close to the outcome event.

The model incorporated MELDNa score and Elixhauser comorbidities obtained significantly higher predictive ability compared to the MELDNa score model. This indicated that to increase predictive probability of mortality during hospitalization, physicians should not only consider the MELDNa score but also presence of comorbidities. Further research is required to develop summary score and cut-off value to predict individual patient’s outcome.

### Limitations

Our study has several limitations. First, we used data derived from one hospital EMRs, and the generalizablility of the results may be a concern. However, generally the c-statistics of the compared risk adjustment methods were consistent with results from other studies in existing literatures [9–12, 15, 20–23]. Second, we only analyzed inpatient EMR data and were unable to assess patients’ outcome after discharge. Third, the odds ratios of certain predictors were not reliable due to low prevalence. The possible reason for the low prevalence of these diseases is that the data is from a hospital specialized in liver disease. However, the purpose of this study is to compare the performance of the common risk adjustment instruments. Lastly, the missing values on certain variables were common. In our EMRs, presence of ascites was well recorded but degree of ascites was often missing (more than 50%). Exclusion of these patients from the CTP score model could under-estimate the c-statistic. Other study also reported that severity of HE and ascites was commonly missing [22].

## Conclusion

The liver specific scoring instruments of CTP and MELDNa outperformed the ECI and CCI methods for predicting in-hospital mortality among patients with cirrhosis using Chinese EMRs. Combining severity and comorbidities could improve the statistical power of predicting in-hospital mortality. These risk adjustment methods should be further evaluated for predicting long-term outcomes.

## Abbreviations

5vMELD: Five-variable MELD
CCI: Charlson comorbidity index
CTP: Child-Turcotte-Pugh
ECI: Elixhauser comorbidity index
EMR: Electronic medical record
HE: Hepatic encephalopathy
MELD: Model for end-stage liver disease
MELDNa: MELD sodium
Na: Sodium
PT-INR: International normalized ratio of prothrombin time
